# The economic burden of urinary tract infections in women visiting general practices in France: a cross-sectional survey

**DOI:** 10.1186/s12913-016-1620-2

**Published:** 2016-08-09

**Authors:** M. François, T. Hanslik, B. Dervaux, Y. Le Strat, C. Souty, S. Vaux, S. Maugat, C. Rondet, M. Sarazin, B. Heym, B. Coignard, L. Rossignol

**Affiliations:** 1 Département de médecine générale, Faculté des sciences de la santé Simone Veille, Université Versailles-Saint-Quentin-en-Yvelines, 78180 Montigny le Bretonneux, France; 2 Sorbonne Universités, UPMC Univ Paris 06, INSERM, Institut Pierre Louis d’Epidémiologie et de Santé Publique (IPLESP UMRS 1136), F75012 Paris, France; 3 Hopital universitaire Ambroise Paré AP-HP, 9, avenue Charles-de-Gaulle, 92100 Boulogne-Billancourt, France; 4 Université Versailles-Saint-Quentin-en-Yvelines, 55 Avenue de Paris, 78000 Versailles, France; 5 Faculté de médecine, CHRU, Lille, France; 6 Institut de Veille Sanitaire, 12, rue du Val d’Osne, 94415 Saint-Maurice cedex, France; 7 Département de médecine générale, Faculté de médecine Pierre et Marie Curie, Sorbonne Université, UPMC Univ Paris 06, Paris, France

**Keywords:** Urinary tract infection, Cost of illness, Primary care

## Abstract

**Background:**

Urinary tract infections (UTIs) are among the most common bacterial infections. Despite this burden, there are few studies of the costs of UTIs. The objective of this study was to determine the costs of UTIs in women over 18 years of age who visit general practitioners in France.

**Methods:**

The direct and indirect costs of clinical UTIs were estimated from societal, French National Health Insurance and patient perspectives. The study population was derived from a national cross-sectional survey entitled the Drug-Resistant Urinary Tract Infection (Druti). The Druti included every woman over 18 years of age who presented with symptoms of UTI and was conducted in France in 2012 and 2013 to estimate the annual incidence of UTIs due to antibiotic-resistant Enterobacteriaceae in women visiting general practitioners (GPs) for suspected UTIs.

**Results:**

Of the 538 women included in Druti, 460 were followed over 8 weeks and included in the cost analysis. The mean age of the women was 46 years old. The median cost of care for one episode of a suspected UTI was €38, and the mean cost was €70. The annual societal cost was €58 million, and €29 million of this was reimbursed by the French National Health Insurance system. In 25 % of the cases, the suspected UTIs were associated with negative urine cultures. The societal cost of these suspected UTIs with negative urine cultures was €13.5 million. No significant difference was found between the costs of the UTIs due to antibiotic-resistant *E. coli* and those due to wild *E. coli* (*p* = 0.63).

**Conclusion:**

In the current context in which the care costs are continually increasing, the results of this study suggests that it is possible to decrease the cost of UTIs by reducing the costs of suspected UTIs and unnecessary treatments, as well as limiting the use of non-recommended tests.

**Electronic supplementary material:**

The online version of this article (doi:10.1186/s12913-016-1620-2) contains supplementary material, which is available to authorized users.

## Background

Urinary tract infections (UTIs) are among the most common bacterial infections [1] and affect nearly half of all women at least once in their lives [2]. Women are more affected than men and exhibit two incidence peaks, i.e., early in the period of sexual activity and in the postmenopausal period [3]. Among those aged 18 years and over, 10.8 % of women reported having at least one UTI within the past 12 months [4]. *Escherichia coli (E. coli)* is the most common urinary pathogen and is found in 74 % of outpatient UTIs [5]. Antimicrobial resistance is increasing and varies between countries, and this variation is strongly related to antibiotic prescription practices [6–9]. Initial *E. coli* UTI episodes are followed in 44 % of cases by recurrence within 12 months [10].

Despite this burden, few studies have examined the costs of UTIs. In 1997, an American study estimated that the burden of UTIs represented 100,000 hospitalizations, 7 million visits and 1 million admissions to emergency services [11]. In 1995, UTI costs were estimated at $1.6 billion in the USA ($659 million in direct costs and $936 million in indirect costs) [4]. The direct cost per patient has been estimated to be between 112 and 172 dollars [12]. In France, these costs are unknown. The main objective of this study was to calculate the direct and indirect UTI costs (including cystitis and acute pyelonephritis) in women over 18 years of age who visit general practices in France. The secondary objectives were to calculate the costs of suspected UTIs with negative urine cultures and to compare the costs of UTIs due to antibiotic-resistant *E. coli* with those of UTIs due to wild *E. coli*.

## Methods

### Population

The data were collected during the Drug Resistance in Community Urinary Tract Infection (Druti) survey. The Druti was a national cross-sectional survey that was conducted in France between January 2012 and February 2013 by general practitioners (GP) of the Sentinelles network [13]. The aim of this survey was to estimate the annual incidence of UTIs due to antibiotic*-*resistant bacteria in women who visited GPs for suspected UTIs [14].

A two-stage sampling design that has been described elsewhere [14] was applied. The eligible patients were females over 18 years old who presented within the previous 7 days with at least one of the following symptoms: dysuria and frequent or urgent of urination (Additional file 1). The patients who agreed to participate and had not taken antibiotics within the prior 7 days were included.

### Data available

For each patient, a urine sample was collected, and urine cultures were performed on all samples in the same laboratory. The urine samples were analyzed, and the antimicrobial susceptibilities were tested. The bacteriological methods are described elsewhere [15, 16]. The GPs were blinded to the urine culture results. When needed, the GPs prescribed another urine culture.

The included patients completed an inclusion questionnaire that contained the patients’ characteristics (i.e., age, clinical status (chronic diseases and comorbidities) and socio-economic data) (Additional file 2). The women completed a questionnaire within 8 weeks following inclusion in which they specified the daily presence or absence of symptoms within the first 14 days (Additional file 3). The women provided information about their health care usage (e.g., physician visits, diagnostic tests, prescription drugs and hospitalization) and sick leave from the baseline time point to 8 weeks. A research assistant contacted by phone the women at two and 8 weeks to collect the data.

### Costs

The direct and indirect costs of clinical UTIs were estimated from the societal perspective, the French National Health Insurance perspective and the patient perspective (prior to private complementary health insurance participation) [17].

French National Health Insurance covers the costs of general and specialized medical visits, prescription drugs, diagnostic tests and hospitalizations. In cases of sickness, the insurance also provides daily allowances for economically active persons, insured and unable to work. Private health insurance can be utilized to reimburse patients for health-related costs that are not covered by social security. For the most disadvantaged, state-run programs provide universal health coverage. The patient contribution corresponds to the costs that are not covered by the French National Health Insurance and the patient’s private health insurance.

Only the costs of the initial UTI episode and associated relapses were taken into account. The costs related to reinfection were not included. The definitions of relapse and reinfection were based on those in the literature [2, 18, 19]. All costs were calculated based on the reported data declared by the women.

The costs are presented in euros. In 2012, the Purchasing Power Parities (i.e., the rates of currency conversion that eliminate the differences in price levels between countries) were $1.1718 and ₤0.8145 for €1 [20].

1. Direct costs
  Direct costs include direct medical costs related to physician visits, diagnostic tests, prescription drugs and hospitalizations [21].
  *Physician visits.* All physician visits were considered including GP visits at baseline. In 2012, the average cost for a physician visit for a woman was determined based on the General Sample of Beneficiaries (EGB), which is permanent representative sample of the population that is protected by the French National Health Insurance [22]. This cost was estimated according to medical specialty and department of residence and was available for the societal, French National Health Insurance and patient perspectives. The French National Health Insurance paid for 70 % of the costs of physician visits.
  *Diagnostic tests*. Only tests performed for UTIs were considered. The costs of the urine cultures that were performed for the incidence study were not included in the analysis. The Nomenclature of Medical Biology Acts (NABM) was used to determine the costs of bio-medical analysis, and the Common Classification of Medical Acts (CCAM) was used to determine the costs of medical imaging procedures. When a patient did not provide the exact title of the diagnostic test, the weighted mean of the cost of same family of investigations (e.g., blood tests or ultrasound) was used. The French National Health Insurance paid for 60 % of the costs of the bio-medical analyses and 70 % of the costs of the medical imaging procedures.
  *Prescription drugs*. Only treatments related to UTIs that were prescribed by a physician and partially (65, 35 or 15 % according to the drug) or totally paid for by the French National Health Insurance were considered. Over the counter drugs dispensed by pharmacists were not taken into account. Two French National Health Insurance databases were used, i.e., the drug’s database (which contains baselines for allopathic medicines that are reimbursed by health insurance) and the MEDICAM (which contains detailed information about reimbursed drugs) [23, 24]. These databases contained the costs of each box of drug (per molecule and by strength, packaging and laboratory), the number of boxes sold and the amount paid by the French National Health Insurance system in 2012. Pediatric and injectable (except third-generation cephalosporin) drugs were removed before the analysis. The average costs weighted by the number of boxes sold in 2012 per molecule and by strength and packaging were calculated. The prescriptions provided the physicians at baseline were used to determine the average cost of a prescription per molecule. This cost was then related to the patient-declared drug consumption.
  *Hospitalizations*. Only admissions related to UTIs were considered. The hospitalizations cost was defined based on the Hospital Stay-Related Group (GHS), which is classification of hospital stays that is based on the care delivered to patients. A tariff order defined by the government was used to determine the cost of the GHS [25]. The GHS were determined based on the patient’s age, disease and medical history [26]. This information was recovered from hospitalization reports that were obtained directly from hospital after acquiring the patient’s consent. The French National Health Insurance reimbursed 80 % of the GHS.
2. Indirect costs
  The indirect costs included only morbidity costs (loss of productivity due to absenteeism) [21]. The friction costs method was used to account for the ability of a company to adapt to the absence of an employee [27]. An elasticity of 0.8 was applied. The daily productivity lost (or gross daily pay) for each women by socio-professional category was obtained by multiplying the gross hourly pay in 2010 based on data from the French National Institute of Statistics and Economic Studies (INSEE) [28] with the number of hours worked per day by a full time equivalent [29]. Next, the employer’s contributions (32.85 % of gross pay) were added [17, 30, 31]. In 2010, the average gross hourly pays were null for non-economically active persons (i.e. students, unemployed person and retired person), €19.42 for manual workers, €21.06 for clerical workers, €29.77 for intermediate occupations and €42.57 for managers.
  The French National Health Insurance pays patient daily allowances that represent 50 % of the gross daily pay [32, 33] only from the fourth day of the sick leave until the end of the sick leave. The daily allowance amounts were also calculated based on the women’s gross hourly pay according to socio-professional category [28]. On the first of January 2012, the daily allowances were capped at €42.77. The patient loss of income was taken as the net daily pay for the first 3 days off, and the difference between the net daily pay and the daily allowances for the following days.

### Economic and statistical analysis

The sampling design (stratification, stages and sampling weights) was taken into account in all of the analyses to make inferences about the population and has been described elsewhere [14]. The average costs of clinical UTIs in France were calculated according to expense items (physician visits, diagnostic tests, prescription drugs, hospitalizations and productivity losses). The total costs according to expense items were calculated by multiplying the average costs by the estimated number of visits to general practices for UTIs in 2012. The mean costs of suspected UTIs that were confirmed and unconfirmed based on urine cultures were compared with Student’s t-tests as were the mean UTIs costs due to wild and antibiotic-resistance *E. coli*.

For the analysis, the *E. coli* were classified as resistant based on disclosed resistance or intermediate susceptibility to a particular antimicrobial agent; otherwise, the isolate was classified as susceptible. Multi-resistance was defined as acquired resistance to at least three classes of antibiotic [34].

The data management and analyses were performed using the R version 2.10.1 software especially the Survey package [35, 36].

## Results

### Population and urine cultures

**Fig. 1:**
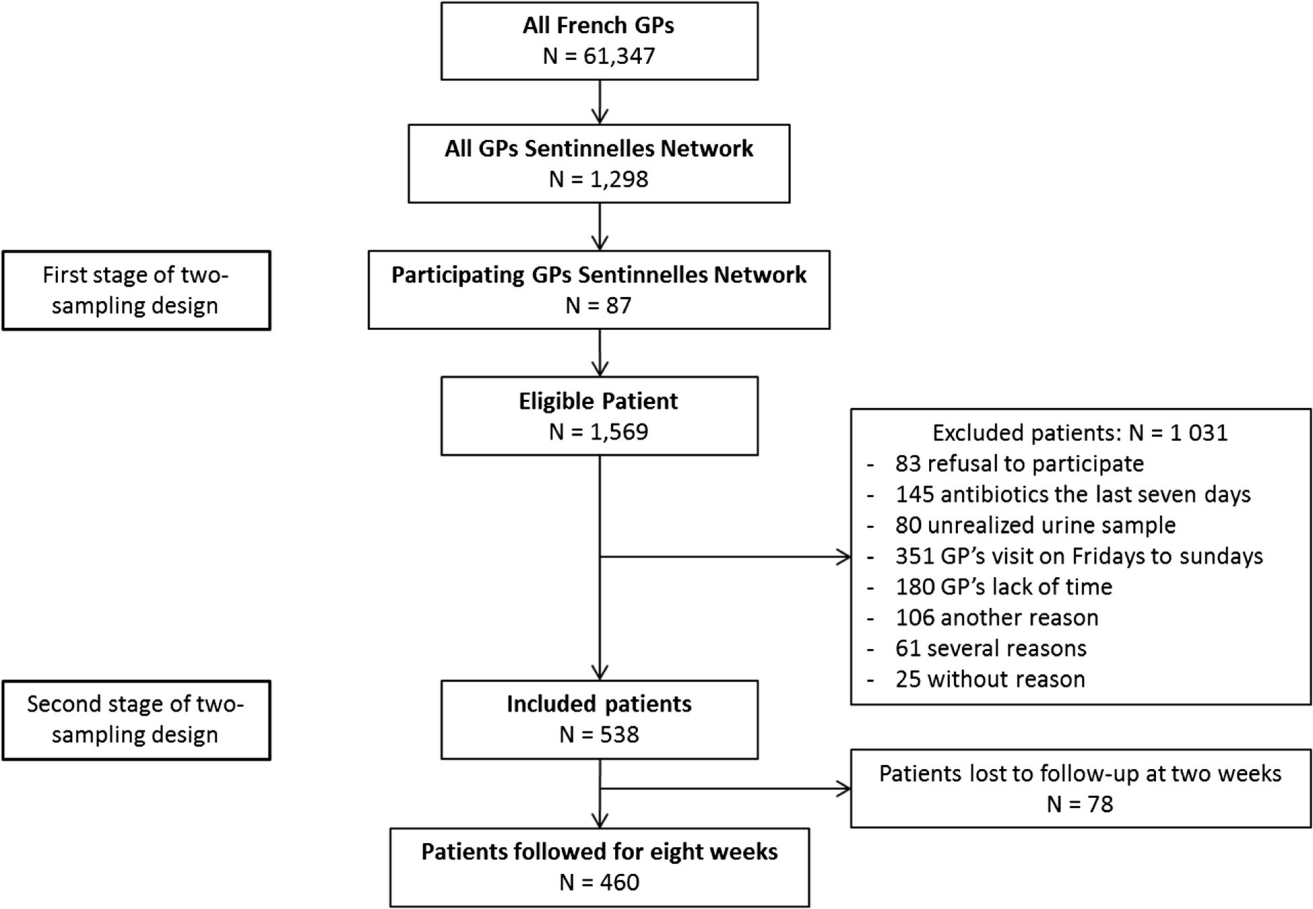
Flow chart

During the study, 87 GPs enrolled 1,569 women who visited with symptoms of UTI. Among these women, 538 were included, and 460 were followed for 8 weeks (Fig. 1). The mean age of the GPs was 53 years (ranging from 33 to 65), and 12 % were women. The mean ages of the eligible women and the women who were followed for 8 weeks were 47 and 46 years, respectively. The women who were followed for 8 weeks had more dysuria and pelvic or flank pain than the eligible women (Table 1). There were more clerical workers and managers and less non-economically active persons in our population than is the general population (Table 2). Of the 460 followed women, 55 remained symptomatic after 2 weeks (12 %).

**Table 1:** Characteristics of the eligible women and the women who were followed for 8 weeks

	Eligible patients *n* = 1,569	Patients followed for 8 weeks, *n* = 460	*p* value^*^
Age (mean, sd)	47 (19)	46 (17)	0.37
Urinary tract infection symptoms (n, %)			
Dysuria	1,431 (91 %)	432 (94 %)	0.08
Frequent urination	1,386 (88 %)	421 (92 %)	0.08
Urinary urgency	1,044 (67 %)	340 (74 %)	0.004
Hematuria	357 (23 %)	121 (26 %)	0.15
Pelvic of lower back pain	582 (37 %)	198 (43 %)	0.031
Fewer	112 (7 %)	32 (7 %)	0.87

^*^chi-square or Student *t*-test

**Table 2:** Socio-economic status of included women and French women in general population

	Percentage of patients followed for 8 weeks, *n* = 460	Percentage for women in general population	*p* value^*^
Manual workers	3.7	5.1	0.17
Clerical workers	33.3	23.5	<0.0001
Intermediate occupations	15	14.9	0.56
Managers	12.6	7.4	<0.0001
Non-economically active persons	35.43	49.1	<0.0001

Complicated UTIs represented 25 % [21–29] of UTI cases. Women treated for a chronic disease (diabetes, cancer or renal insufficiency), pregnancy and urinary tract anomalies represented 6 % [4–8], 2 % [1–3], and 4 % [2–6] of UTI cases, respectively.

The number of visits to general practices for suspected UTIs was estimated to be 823,073 among over the age of 18 years in 2012 (95 % confidence interval (CI): 623,614–1,040,532). Among these clinical UTIs, 626,046 (95 % CI: 465,196–786,896) were confirmed by positive urine cultures, and 518,446 (95 % CI: 381,981–654,911) of these UTIs were due to *E. coli.*

### UTIs costs

#### Physician visits

After inclusion, 14 % (95 % CI: 10–20 %) of the women had further visits with a GP, an urologist or a gynecologist (Table 3). The mean costs per patient were €27.69 (95 % CI: €25.81–29.56) from the societal perspective and €17.44 (95 % CI: €16.27–18.6) from the French National Health Insurance perspective.

**Table 3:** Direct UTI costs in France

		Estimated proportion of patients consuming at least one care modality^c^	Mean cost per care modality^c^	Societal cost (millions €)	French National Health Insurance cost (millions €)	Patient dost (before private complementary millions €)
All visits	GP (after inclusion)	13 % (9–19 %)	23,72 (23,67–23,77)	23.0	14.5	8.5
	Gynecologist	1 % (0.4–3 %)	40,68 (39,93–41,44)			
	Urologist	0.4 % (0.06–2 %)	56,71 (52,16–61,26)			
Diagnostic tests	Blood test	2.5 % (1–5 %)	13,50 (11,38–15,64)	9.6	6.0	3.6
	Urine culture	24 % (18–31 %)	17,55^d^			
	Ultrasound	8 % (5–12 %)	67,29 (63,84–70,73)			
	Urology scan	0.3 % (0.1–2 %)	150,77			
Prescription drugs	Fosfomycin	39 % (30–49 %)	8.36^d^	10.5	6.1	4.4
	Other antibiotic^a^	64 % (55–73 %)	13.27 (12.37–14.17)			
	Analgesics	13 % (10–17 %)	3.98 (3.70–4.27)			
	Other prescriptions^b^	4 % (2–8 %)	5.17 (4.39–5.95)			
Hospitalization		0.06 % (0.01–1.2 %)	1294.67	0.9	0.7	0.2

Sources: General Sample of Beneficiaries (EGB), Nomenclature of Medical Biology Acts (NABM) and Common Classification of Medical Acts (CCAM), drug base (base de medicaments) and MEDICAM

*€* euros

^a^amoxicillin, amoxicillin – clavulanic acid, cefixime, cefpodoxime, ceftriaxone, ciproloxacine, enoxacine, lomefloxacin, nirofurantoin, norfloxacin, nystatin, oxfloxacin, pefloxacin, nalidixic acid, trimethoprim-sulphamethoxazole

^b^Antifungals, digestive transit regulators, hormonal treatments, proton pump inhibitors with non-steroidal anti-inflammatory drugs and corticosteroids

^c^Estimated proportion or mean cost (95 % CI, CI: confidence interval) with the sampling design

^d^No 95 % CI due to exact cost

#### Diagnostic tests

At least one diagnostic test was performed for 29 % of the women (95 % CI: 22–36 %) including 24 % (95 % CI: 18–31 %) of women who underwent a urine culture and 8 % (95 % CI: 5–12 %) who underwent an ultrasound (Table 3). Nine percent (95 CI: 0–19 %) of women who underwent an ultrasound had a history of urinary tract abnormality. The mean costs per patient were €11.54 (95 % CI: €8.81–14.26) from the societal perspective and €7.27 (95 % CI: €5.54–8.99) from the French National Health Insurance perspective. Among these tests, 52 % (95 % CI: 43–60 %) were performed outside of the French recommendations.

#### Prescription drugs

Antibiotics were prescribed to 98 % (95 % CI: 96–99 %) of the women. Other treatments (i.e., analgesics, antifungals, digestive transit regulators, hormonal treatments, proton pump inhibitors with non-steroidal anti-inflammatory drugs and corticosteroids for cases with antibiotic allergies) were prescribed to 17 % (95 % CI: 13–22 %) of the women (Table 3). The mean costs per patient were €12.68 (95 % CI: €11.53–13.83) from the societal perspective and €7.30 (95 % CI: €6.60–8.01) from the French National Health Insurance perspective.

#### Hospitalization

Only one patient in this study was hospitalized for pyelonephritis. This hospitalization cost was €1240.67 (Table 3). The mean costs were patient was €1.13 (95 % CI: €0–3.28) from the societal perspective and €0.86 (95 % CI: €0–2.51) from the French National Health Insurance perspective.

#### Indirect costs

Nine percent of the women (95 % CI: 7–13 %) took sick leaves. Among these women, 15 % (95 % CI: 6–32 %) took sick leaves longer than 3 days and received daily allowances from the French National Health Insurance. The mean sick leave duration was 2.39 days (95 % CI: 1.62–3.15) (Table 4). The mean costs per patient were €16.71 (95 % CI: €8.79–24.63) from the societal perspective and €1.63 (95 % CI: €0–3.34) from the French National Health Insurance perspective.

**Table 4:** Morbidity costs: Loss of productivity due to absenteeism due to urinary tract infection (UTI) in France

	Societal perspective	French National Health Insurance perspective		
	Estimated proportion of patients with sick leave (% (95 % CI))^a^	Sick leave daily cost (euros)	Estimated proportion of patients with sick leave longer than 3 days	Daily allowances daily cost (euros)
Women visiting GP for suspected UTI	9 % (7–13 %)		1 % (0.6–3 %)	
−Manual worker	0.3 % (0.04–2 %)	76.89	0.05 % (0.01–0.1 %)	29.21
−Clerical workers	5 % (3–8 %)	82.73	1 % (0.03–3 %)	31.78
−Intermediate occupations	3 % (1–5 %)	116.89	0.1 % (0.2–1 %)	42.77
−Managers	1 % (0.3–2 %)	188.91	0.1 % (0.02–1 %)	42.77
Total cost (million euros)	13.9	1.4		

Source: French National Institute of Statistics and Economic Studies (INSEE)

^a^estimated proportion with the sampling design and 95 % CI; CI: confidence interval

Overall in France in 2012, the mean global cost for a suspected UTI episode was €69.73 (95 % CI: €58.54–€80.92) for women over 18 years of age who visited a GP, and the median cost was €37.74 (Table 5). Based on the estimation of 823,073 visits for UTI views in general practices in 2012, the annual total cost of suspected UTIs was €58 million.

**Table 5:** Mean urinary tract infection costs per patient from the perspectives (in euros)

	Societal perspective	French National Health Insurance perspective	Patients perspective^a^
Mean cost (95 % confidence interval)	70 (59–81)	34.50 (31–38)	34 (27–42)
25th percentile	31.68	19.75	11.73
50th percentile	37.74	23.58	15.10
75th percentile	60.86	32.52	24.34
Total cost (million euros)	58	29	29

^a^Before private complementary reimbursement

### Costs of suspected UTIs with negative urine cultures

Among the urine cultures, 75 % (95 % CI: 70–79 %) were positive. *E. coli* was the most common pathogen (77.4 %; 95 % CI: 73–81 %). The care consumptions were similar among women with positive and negative urine cultures. The mean cost per patient did not significantly differ between the two groups, i.e., €70.96 (95 % CI: €58.99–82.92) for the women with positive urine cultures and €66.13 (95 % CI: €48.39–83.87) for the women with negative urine cultures (*p* = 0.60). From the societal perspective, the total cost of suspected UTIs with negative urine cultures was €13.6 million (23 % of the total UTI costs).

### Mean costs of UTIs due to antibiotic-resistant *E. coli* and wild *E. coli*

Among the *E. coli*-positive urine cultures, 38 % (95 % CI: 31–45 %) were resistant to at least one antibiotic, and 19 % (15–24 %) were multi-resistant. The care consumptions were similar for the women infected with resistant and wild *E. coli.* The mean cost per patient for UTIs due to wild *E. coli* was €74.76 (95 % CI: €57.61–91.91), which did not significantly differ from the mean UTI cost due to *E. coli* strains that were resistant to at least one antibiotic (€67.44; 95 % CI: €43.93–90.95; *p* = 0.63) or the mean UTI cost due to multi-resistant *E. coli* (€74.49; 95 % CI: €30.87–118.11; *p* = 0.99)*.*

## Discussion

This initial study conducted in France on the costs of community UTIs in women over 18 years of age estimated a total cost from the societal perspective of €58 million in 2012, €44 million for direct costs and €14 million for indirect costs. Half of this cost was supported by the French National Health Insurance, and half was supported by the patients (before private complementary health insurance participation). Visits represented the largest expense item followed by sick leave and prescription drugs. Although very expensive, hospitalizations were rare and therefore represented the smallest expense item. The costs for 75 % of the women were below the mean cost. Additional visits with specialist physicians, ultrasounds, hospitalizations and sick leave concerned less than one quarter of the women. The important costs of these additional health care procedures clearly increased the mean UTI cost.

An important strength of this study was the use of a sampling design. This allowed to correct the bias due to drop-outs and geographical repartition and to generalize with caution the cost of UTIs to the population of women over 18 years of age who visit GPs for presumed UTIs. Furthermore, to estimate the possible costs according to expense items as accurately as possible, the maximum amounts of data from the French National Health Insurance and the French National Institute of Statistics and Economic Studies were used. The use of the EGB allowed for the accounting of possible differences in care consumption according to gender and excess fees according to medical specialties and French departments, particularly in terms of the costs of physician visits [37]. The systematic collection of urine sample from all women who visited their GPs for suspected UTIs permitted the distinction between clinical UTIs with positive urine cultures from clinical UTIs with negative urine cultures and the estimation of their related costs. Furthermore, to estimate the real cost, the women’ declarations were preferred to the GPs’ declaration to account only for prescriptions that were actually utilized or purchased.

This study has some limitations that might have resulted in the over- or underestimation of the costs of UTIs. First, the cost generalization should be interpreted with caution: women suffering from UTIs without dysuria, frequent or urgent of urination were not included, which could decrease the estimated cost of UTIs; patients included had more symptoms than eligible patients, which could influence the achievement of diagnostic test, especially lower back pain with suspected pyelonephritis; the socio economic status was not available for eligible women, preventing to compare eligible and included patients on this point. However, in our study, there were more workers and less non-economically active persons than in the general population. This is concordant with the French inequalities of health care recourse: unemployed and retired persons seek less care than economically active persons [38]. Second, the costs calculated in this study were for women who visited GPs and not for the general population. The estimation of the costs of UTIs among the general population would have required the estimations of the costs of women who visited hospital emergencies departments and specialists (i.e., urologists and gynecologists). Third, the data used to estimate the non-medical direct costs (i.e., time lost and monetary expenses), intangible costs (i.e., loss of well-being for the patient and her close family and friends) and presenteeism costs (i.e., the loss of productivity due to a UTI while the patient was working) were not available. Fourth, the friction cost method was chosen to determinate the indirect costs. The cost’s results should be interpreted with caution because this method is controversial in cases of short-term disease. The human capital approach overstates the production lost because the sick employee’s colleagues can offset the absence via increased productivity [27]. Consequently, the estimates of friction costs should represent the upper bound of the estimates of the short-term indirect costs [27]. However, in cases involving teamwork, the absence of an employee can also reduce the production of several employees [39]. Fifth, although the study was designed to exclude the costs of reinfection, the women who were symptomatic at 2 weeks might have experienced a reinfection between the second and the eighth weeks, and these costs were taken into account because differences between relapses and reinfections could not be identified during this period. Sixth, the costs of long-term symptomatic failure (i.e., the persistence of symptoms after 8 weeks) could not be taken into account because the follow-up period stopped after 8 weeks. Seventh, from the patient perspective, the costs could have been overestimated because some companies might have paid their employees during their sick leaves; or these costs could also have been underestimated because there were no data from which excess fees for medical imaging procedures could be assessed. Another point from the patient perspective is the costs of over the counter drugs, which could not still have been accounted for because costs differ between retail outlets. None of these data were available. As last limitation, the results of diagnostic tests prescribed by physicians were not collected.

According to Foxman [4], 10.8 % of women over 18 years of age experience at least one UTI per year, which represents more than 2.5 million people in France as of 1 January 2012 [40]. In our study, the estimated number of women who visited a GP for a UTI was estimated to 832,073 (95 % CI = 623,614–1,040,532) in 2012. However, the rate of care seeking for UTIs in France is unknown. Some women with UTI symptoms might have spontaneously recovered healed [41], visited other specialists (e.g., gynecologists and urologists) or emergency departments. The yearly number of emergency visits for UTIs is estimated to 410 000 (2.3 % of the 18 million of emergency visits), half less than in primary care [42, 43].

In Italy, the mean annual direct cystitis cost (i.e., physician visits, diagnostic tests and prescription drugs) from the Italian National Health Service (NHS) perspective was evaluated to be €229 per patient between January 2007 and December 2010 [44]. Each patient had an average of 4.5 episodes per year. The cost for the Italian NHS was higher than the cost for the French National Health Insurance. However, the women included in the Italian study visited referral centers for the treatment of cystitis, which might have resulted in the selection of more complicated UTIs. In the United Kingdom, a cost analysis was performed to evaluate the mean monthly direct UTIs costs in women between the ages of 18 and 70 years who were seen by a GP or a nurse between 2005 and 2006 from the NHS perspective. The mean monthly direct UTI costs were estimated at £30.60 when the UTIs were treated with empirical antibiotic therapy and £37.10 when urine cultures were performed [45]. The cost differences between this study and the present French study may have been related to the systematic one-month follow-up (with no differences between relapse and reinfection) used in the English study and the higher remuneration of English GPs (approximately 30 % greater than that of French GPs in 2008 [46]). In the United States in 2010, the annual direct and indirect UTI cost was estimated at $2.3 billion [47]. Considering that the American population was approximately five times greater than the French population in 2012, the American cost was six times greater than the French cost. This difference could be explained by the costs of physician visits and diagnostic tests, which are three to ten times more expensive in the United States than in France [48]. Cost-effectiveness studies have found that most cost-effective treatment is the empirical use of antibiotics that are effective against *E. coli* [12, 49, 50]. Furthermore, in the present French study, the mean UTI cost due to wild *E. coli* was not significantly different from the mean UTI cost due to antibiotic-resistant *E. coli*. This lack of a difference could have resulted from our use of systematic urine analyses. Even without antibiotics, 20 % of women recover from uncomplicated lower UTIs within 3 days and 26 % recover within one week [41, 51]. The effect of inadequate antibiotic prescription should be studied in greater detail and might have little influence on the consumption of care by women who visit GPs for UTIs.

Approximately one quarter of the suspected UTIs seen in general practice had negative urine cultures. This illustrates the limits of the clinical diagnosis of urinary tract infections. The probability of having an UTI for a woman with urinary symptoms is 48 % [52]; this probability increases if dysuria, urinary frequency or hematuria is present [53]. This suspected UTIs with negative urine cultures had a significant influence on cost, i.e., more than 23 % of the overall UTI cost. The environmental influence of antibiotic therapy for these women cannot be neglected due to the risk of the selection of resistant bacteria [54]. More than half of diagnostic tests performed for UTIs were prescribed outside of the recommendations as previously reported by other authors [55]. It is interesting to consider how to the prescription of potentially unnecessary and environmentally harmful treatments the administration of potentially inappropriate diagnostic tests can be prevented.

## Conclusions

In the current economic context in which the costs of care are continually increasing, the present study estimated that the cost of UTIs among women who visited their GPs was €58 million from the societal perspective, and half of this value was reimbursed by the French National Health Insurance. This study provides new perspectives regarding the possibility of reducing the cost of the management of this pathology without reducing the quality of care, particularly via the prescription of diagnostic tests. For women with negative urine cultures, the development of new effective diagnostic tools could reduce antibiotic prescriptions and the costs of these UTIs. Another study should be performed to estimate the total UTI costs in France that includes the costs of women who visit hospital emergencies departments and specialists (e.g., urologists and gynecologists).

## Abbreviations

CCAM, common classification of medical acts; EGB, general sample of beneficiaries; GHS, hospital stay-related group; GP, general practitioners; CI, confidence interval; INSEE, French National Institute of Statistics and Economic Studies; NABM, nomenclature of medical biology acts; NHS, national health service; UTI, urinary tract infection

## Additional files

Additional file 1: First page of register for each doctor. (DOC 97 kb) Additional file 2: Inclusion questionnaire. (DOC 195 kb) Additional file 3: Follow-up questionnaire. (DOC 108 kb)
