# Supplementary material for: The economic burden of urinary tract infections in women visiting general practices in France: a cross-sectional survey
Source: BMC Health Serv Res. 2016 Aug 9;16:365. doi: 10.1186/s12913-016-1620-2 (PMC4977873; doi:10.1186/s12913-016-1620-2)
Supplement: Additional file 1: — First page of register for each doctor. (DOC 97 kb) [file 12913_2016_1620_MOESM1_ESM.doc]

# Druti – Register

| **Num** | **Age** | **Date of visit** | **Symptoms** | | | **Inclusion** | **Reason for non-inclusion**  **(thank you to check a box)** | **Inclusion** | **Date of post** | **Name and first name**  **+ unique id** |
| --- | --- | --- | --- | --- | --- | --- | --- | --- | --- | --- |
|  |  |  |  | **Yes** | **No** |  |  |  |  |  |
| 1 | _ _ | _ _ / _ _ / _ _ | Pain or bladder tenderness | ❒ | ❒ | ❒ No | ❒ **antibiotics in the last 7 days**  ❒ prior prescription ❒ recent prescription  ❒ patient refusal  ❒ Unrealized urine sample  ❒ consulting days do not allow a sending in 48 hours (Fri and Sat and Thu and more if Corse)  ❒ lack of time / too many patients  ❒ other : ………………………………………… | **❒ Yes** | _ _/_ _/_ _ _ _ | ______________  ______________  /_ _ _ _/- /_ _/ |
|  |  |  | Pollakiuria | ❒ | ❒ |  |  |  |  |  |
|  |  |  | Urinary urgency | ❒ | ❒ |  |  |  |  |  |
|  |  |  | Hematuria | ❒ | ❒ |  |  |  |  |  |
|  |  |  | Pelvic or lumbar pain | ❒ | ❒ |  |  |  |  |  |
|  |  |  | Fever (>38,5°Celsius) | ❒ | ❒ |  |  |  |  |  |
|  |  |  | Pruritus / vaginal discharge | ❒ | ❒ |  |  |  |  |  |
| 2 | _ _ | _ _ / _ _ / _ _ | Pain or bladder tenderness | ❒ | ❒ | ❒ No | ❒ **antibiotics in the last 7 days**  ❒ prior prescription ❒ recent prescription  ❒ patient refusal  ❒ Unrealized urine sample  ❒ consulting days do not allow a sending in 48 hours (Fri and Sat and Thu and more if Corse)  ❒ lack of time / too many patients  ❒ other : ………………………………………… | **❒ Yes** | _ _/_ _/_ _ _ _ | ______________  ______________  /_ _ _ _/- /_ _/ |
|  |  |  | Pollakiuria | ❒ | ❒ |  |  |  |  |  |
|  |  |  | Urinary urgency | ❒ | ❒ |  |  |  |  |  |
|  |  |  | Hematuria | ❒ | ❒ |  |  |  |  |  |
|  |  |  | Pelvic or lumbar pain | ❒ | ❒ |  |  |  |  |  |
|  |  |  | Fever (>38,5°Celsius) | ❒ | ❒ |  |  |  |  |  |
|  |  |  | Pruritus / vaginal discharge | ❒ | ❒ |  |  |  |  |  |
| 3 | _ _ | _ _ / _ _ / _ _ | Pain or bladder tenderness | ❒ | ❒ | ❒ No | ❒ **antibiotics in the last 7 days**  ❒ prior prescription ❒ recent prescription  ❒ patient refusal  ❒ Unrealized urine sample  ❒ consulting days do not allow a sending in 48 hours (Fri and Sat and Thu and more if Corse)  ❒ lack of time / too many patients  ❒ other : ………………………………………… | **❒ Yes** | _ _/_ _/_ _ _ _ | ______________  ______________  /_ _ _ _/- /_ _/ |
|  |  |  | Pollakiuria | ❒ | ❒ |  |  |  |  |  |
|  |  |  | Urinary urgency | ❒ | ❒ |  |  |  |  |  |
|  |  |  | Hematuria | ❒ | ❒ |  |  |  |  |  |
|  |  |  | Pelvic or lumbar pain | ❒ | ❒ |  |  |  |  |  |
|  |  |  | Fever (>38,5°Celsius) | ❒ | ❒ |  |  |  |  |  |
|  |  |  | Pruritus / vaginal discharge | ❒ | ❒ |  |  |  |  |  |
