# Supplementary material for: The economic burden of urinary tract infections in women visiting general practices in France: a cross-sectional survey
Source: BMC Health Serv Res. 2016 Aug 9;16:365. doi: 10.1186/s12913-016-1620-2 (PMC4977873; doi:10.1186/s12913-016-1620-2)
Supplement: Additional file 2: — Inclusion questionnaire. (DOC 195 kb) [file 12913_2016_1620_MOESM2_ESM.doc]

**Druti: medical questionnaire**

Doctor name and first name: Department of practice:

Doctor’s stamp Unique id

**1. Is the information about patient symptoms reported in the registry?**

Yes  No 

Otherwise, thank you to complete because these information are essential to the study.

**Case description**

2. Date of symptoms onset _ _ / _ _ / _ _ _ _ (day / month / year)

3. Risk of complicated urinary tract infection:

| Urinary tract anomalies | No  | Yes  |
| --- | --- | --- |
| Pregnancy | No  | Yes  |
| Chronic disease  (for example : diabetes, immunosuppression, renal failure) | No  | Yes  |
| If yes, describe______________________________________________  __________________________________________________________________ | | |

4. Did you perform a dipstick: No Yes 

5. If yes, what was the result (**- number of +**)?

Leukocyturia: Nitrite: Hematuria:

**Diagnostic probability**

| 6. Do you bet that the urine analysis is positive? | Yes  No  |
| --- | --- |
| 7. Did the patient already have a urinary tract infection in the past? | Yes  No  |
| 8. Do the patient bet that she is suffering from a urinary tract infection? | Yes  No  |

**Support for the current episode**

9. Did the patient take a treatment for this episode before consultation: No  Yes 

10. If yes, describe_____________________________________________________________

11. Have some diagnostic tests been performed: No  Yes 

12. If yes, please select check boxes

urine analysis (in addition to that of the study) 

ultrasound , Which localization: __________________________________

other diagnostic tests  If yes, describe: ________________________________________

13. Treatment prescribed during this consultation:

| Treatment name | Dosage | Length |
| --- | --- | --- |
|  |  |  |
|  |  |  |
|  |  |  |
|  |  |  |

14. Did you prescribe a sick leave?

No  Yes  14 bis. If yes, length: _ _ days

15. Hospitalization following the consultation:

No  Yes 

**Patient characteristics**

16. To your knowledge, did the patient have a urinary tract infection in the last 12 months?  (Do only take into acount episodes older than 8 weeks)

No  Yes 

17. **If yes**, number of urinary tract infection during the last 12 months: _ _

18. To your knowledge, has the patient received antibiotic treatment (regardless of indication) during the last 3 months?

No  Yes  **If yes, please complete table 1.**

19. To your knowledge, has another household member received antibiotics (regardless of indication) during the last 3 months?

No  Yes 

20. **If yes**, number of antibiotic treatment for all household members during the last 3 months: _ _

21. During the last 12 months, has the patient been hospitalized (regardless of indication, and length of stay)?

No  Yes   **If yes, please complete table 2.**

22. **If yes**, number of hospitalizations during the last 12 months: _ _

23. During the last 3 months, did the patient have physician visits?

No  Yes 

24. **If yes**, number of physician visits during the last 3 months: _ _

25. During **the** **last** **month**, did the patient have contact with a person hospitalized or institutionalized?

No  Yes 

26. **If yes**, thank you to specify the following information: (one or more answers)

Occasional private contact 

Repeated business contact (health professional for example) 

27. To your knowledge and during **the** **last** **month**, one member of the patient household has had contact with a person hospitalized or institutionalized?

No  Yes 

28. **If yes**, thank you to specify the following information: (one or more answers)

Occasional private contact 

Repeated business contact (health professional for example) 

29. During **the** **last** **month**, did the patient have an urinary catheterization?

No  Yes 

30. **If yes**, precise:

indwelling catheter 

intermittente catheter 

31. During the **last 12 months**, did the patient travel abroad?

No  Yes 

32. **If yes**, number of travels during the last 12 months: _ _

**And, please complete table 3.**

33. Is the patient aware of a multi resistant germ infection concerning her?

No  Yes  Don’t know 

34. During the **last 12 months**, did the patient host at least one person usually living abroad?

No  Yes 

35. **If yes**, from which country: ________________________

36. Has the patient daily contact with animals?

No  Yes  **If yes, please complete table 4.**

37. Did the patient eat raw meat during the last 3?

No  Yes  Don’t know 

**Table 1: Information on antibiotic**

If you answered yes to question 18, thank you to complete the table

| Antibiotic treatment n° | 1 | 2 | 3 | 4 | 5 | 6 |
| --- | --- | --- | --- | --- | --- | --- |
| Start date | _ _ /_ _/_ _ | _ _ /_ _/_ _ | _ _ /_ _/_ _ | _ _ /_ _/_ _ | _ _ /_ _/_ _ | _ _ /_ _/_ _ |
| Antibiotic |  |  |  |  |  |  |
| Length (select correct check box) |  ≤ 3 days   4 à 7 days   ≥ 8 days |  ≤ 3 days   4 à 7 days   ≥ 8 days |  ≤ 3 days   4 à 7 days   ≥ 8 days |  ≤ 3 days   4 à 7 days   ≥ 8 days |  ≤ 3 days   4 à 7 days   ≥ 8 days |  ≤ 3 days   4 à 7 days   ≥ 8 days |

**Table 2: Information on hospitalizations**

If you answered yes to question 21, thank you to complete the table

| Hospitalization n° | 1 | 2 | 3 | 4 | 5 | 6 |
| --- | --- | --- | --- | --- | --- | --- |
| Start date | _ _ /_ _/_ _ | _ _ /_ _/_ _ | _ _ /_ _/_ _ | _ _ /_ _/_ _ | _ _ /_ _/_ _ | _ _ /_ _/_ _ |
| Length |  |  |  |  |  |  |
| Passage in intensive care | Yes   No   NPH*  | Yes   No   NPH*  | Yes   No   NPH*  | Yes   No   NPH*  | Yes   No   NPH*  | Yes   No   NPH*  |

***** Not Pronounced Herself

**Tableau 3: Information on travels.**

If you answered yes to question 31, thank you to complete the table

| Travel n° | 1 | 2 | 3 | 4 | 5 | 6 |
| --- | --- | --- | --- | --- | --- | --- |
| Country |  |  |  |  |  |  |
| Length |  |  |  |  |  |  |
| Departure date (month/year) | _ _ /_ _ _ _ | _ _ /_ _ _ _ | _ _ /_ _ _ _ | _ _ /_ _ _ _ | _ _ /_ _ _ _ | _ _ /_ _ _ _ |
| Contact with the country's health system | Yes   No  | Yes   No  | Yes   No  | Yes   No  | Yes   No  | Yes   No  |
| If yes, precise |  |  |  |  |  |  |
| Planned hospital stay | Yes   No  | Yes   No  | Yes   No  | Yes   No  | Yes   No  | Yes   No  |
| Emergency hospitalization | Yes   No  | Yes   No  | Yes   No  | Yes   No  | Yes   No  | Yes   No  |
| Dialysis | Yes   No  | Yes   No  | Yes   No  | Yes   No  | Yes   No  | Yes   No  |
| Passage in intensive care | Yes   No  | Yes   No  | Yes   No  | Yes   No  | Yes   No  | Yes   No  |

**Tableau 4: Information on animals.**

**If you answered yes to question 21, thank you to complete the table, precise for each kind of animals**

| Kind of animals |  |  |  |  |  |
| --- | --- | --- | --- | --- | --- |
| Number |  |  |  |  |  |
| Contact Type | Professional   Pet  | Professional   Pet  | Professional   Pet  | Professional   Pet  | Professional   Pet  |
| Has one of these animals received antibiotics during the last three months | Yes   No   NPH*  | Yes   No   NPH*  | Yes   No   NPH*  | Yes   No   NPH*  | Yes   No   NPH*  |

***** Not Pronounced Herself

**Caution section on socioeconomic characteristics on page 4. Please turn the page.**

**Socioeconomic characteristics**

*This part can be filled directly by the patient, while the doctor packs the urine sample.*

We thank you for your participation in our study. To complete the answers you provided to your doctor, thank you to answer the following questions to assess the economic impact of urinary tract infections and describe the social characteristics of patients concerned. Your responses will remain strictly confidential and anonymous.

| 38. Do you live alone? | Yes   No   If not, how many people including yourself, are there in your home: __ |
| --- | --- |
| 39. What is your current occupation? | Employed   Trainee / Internship   Pupil / Student   Unemployed   Retired  Precise the last employ : ___________  Home   Parental leave full time   Others  Precise: ____________________ |
| 40. What is your socio economic status? | Farmer   Artisan / Retail trader / Company head   Executive / Intellectual profession   Intermediate profession   Worker   Labor   Non-working  |
| 41. What is your education level? | Never attended school   Infant school / primary school   Secondary school   Higher education  |
| 42. What is your nationality? | French   Other  Precise: ________________________ |
| 43. What is the parents’ nationality? | French for both parents   Other nationality for at least one parent  |
| 44. What is your zip code? |  |
| 45. Currently health coverage? | Standard health insurance   Universal health care coverage   State Medical Assistance   No health coverage   I don’t know  |
| 46. Extended health coverage? | Yes by Universal health care coverage   Yes by mutual or private insurance   Yes but I don’t know why   None   I don’t know  |

**Thank you for answering.** We will keep your doctor informed of the progress of the study. Do not hesitate to contact him or us if you would like any additional information.
