# Supplementary material for: The economic burden of urinary tract infections in women visiting general practices in France: a cross-sectional survey
Source: BMC Health Serv Res. 2016 Aug 9;16:365. doi: 10.1186/s12913-016-1620-2 (PMC4977873; doi:10.1186/s12913-016-1620-2)
Supplement: Additional file 3: — Follow-up questionnaire. (DOC 108 kb) [file 12913_2016_1620_MOESM3_ESM.doc]

**Druti follow-up questionnaire** (to be given to the patient, whether she accepts)

Doctor name and first name: Department of practice:

Doctor’s stamp Unique id:

To prepare these calls, could you keep information about your medical expenses (drugs, prescriptions, hospitalization or diagnostic test reports)

**Evolution of symptoms the first two weeks**

1. Thank you to specify daily changes in your symptoms during the two weeks after the consultation.

| Day after your first consultation | 1 | 2 | 3 | 4 | 5 | 6 | 7 | 8 | 9 | 10 | 11 | 12 | 13 | 14 |
| --- | --- | --- | --- | --- | --- | --- | --- | --- | --- | --- | --- | --- | --- | --- |
| Are you still disturbed? | Yes   No  | Yes   No  | Yes   No  | Yes   No  | Yes   No  | Yes   No  | Yes   No  | Yes   No  | Yes   No  | Yes   No  | Yes   No  | Yes   No  | Yes   No  | Yes   No  |

**Interview at 2 weeks**

2. Your doctor had he prescribed diagnostic tests during your first consultation?

No  Yes 

3. If yes, which one? .................................

4. Did you purchase the medication prescribed by your doctor during your first consultation?

No  Yes 

5. If yes, which one? .................................

6. If your doctor prescribed an antibiotic, did you take all of your treatment?

No  Yes 

7. For this urinary problem, did you take other treatments than those prescribed by your doctor, including over the counter drugs?

No  Yes 

8. If yes, which one? .................................

9. For this urinary problem, did you need to visit a doctor again?

No  Yes 

10. If yes, thank you to complete the following table:

| Doctor specialty (general practitioner/other specialty) | Number of visits  (0 if none) |
| --- | --- |
| The general practitioner saw at the first consultation | _ _ |
| Another general practitioner | _ _ |
| Doctor with another specialty  Which specialty: ........................... | _ _ |

10 bis. Has the doctor prescribed medication? No  Yes 

If yes, which one did you purchase?

11. For this urinary infection from the initial consultation, was there a need to perform non-prescribed diagnostic tests at the initial consultation?

No  Yes 

12. If yes, which one? .................................

13. Have you been hospitalized since the first consultation?

No  Yes 

14. If yes, thank you to specify the dates, service and the reasons for hospitalization: ..................................................................

..................................................................

15. For this urinary problem, you missed one or more days of work?

No  Yes 

16. If yes, how many days: __ __

17. And have you benefited from a sick leave by your doctor? No  Yes 

18. If yes, how many days: __ __

**Interview at 8 weeks**

19. Over the past 6 weeks, have you had new urinary symptoms?

No  Yes 

**If you answered no to question 19, thank you to respond directly to question 31.**

**If you answered yes to question 19, thank you to answer the following questions.**

20. Have you nedd to visit a doctor for these new signs?

No  Yes 

21. If yes, thank you to complete the following table:

| Doctor specialty (general practitioner/other specialty) | Number of visits  (0 if none) |
| --- | --- |
| The general practitioner saw at the first consultation | _ _ |
| Another general practitioner | _ _ |
| Doctor with another specialty  Which specialty: ........................... | _ _ |

22. For this urinary problem, did you take other treatments than those prescribed by your doctor, including over the counter drugs?

No  Yes 

23. If yes, which one? ................................

24. Was it necessary to practice new diagnostic tests for these new urinary symptoms?

No  Yes 

25. If yes, which one? ................................

26. Have you been hospitalized because of these new urinary symptoms?

No  Yes 

27. If yes, thank you to specify the dates, service and the reasons for hospitalization: ..................................................................

..................................................................

28. Have you missed one or more days of work because of these new urinary symptoms?

No  Yes 

29. If yes, how many days: __ __

29. And have you benefited from a sick leave by your doctor?

No  Yes 

30. If yes, how many days: __ __

**31. Would you agree to be contacted within 12 months in order to continue our research on urinary tract infections?**

No  Yes 
